# A Multi-Modal Few-Shot Learning Framework for Foreign Object Segmentation in GIS Inspection

**DOI:** 10.3390/s26092911

**Published:** 2026-05-06

**Authors:** Jiaxin Liu, Yexing Lang, Jianeng Tang, Qiang Li, Songting Yang, Songyi Dian

**Affiliations:** 1 State Grid Liaoning Electric Power Research Institute, Shenyang 110006, China; liujxldk@163.com (J.L.); bianyushan_ever@163.com (Y.L.); jialuntang@126.com (J.T.); 2 State Grid Liaoning Electric Power Co., Ltd., Shenyang 110004, China; 3 Sichuan Lecheng Electric Technology Co., Ltd., Chengdu 611730, China; liqiangcqcs@163.com; 4 College of Electrical Engineering, Sichuan University, Chengdu 610065, China; 18030957571@163.com

**Keywords:** GIS, few-shot foreign object segmentation, multi-modal image fusion, MSBNet

## Abstract

The reliable operation of Gas-Insulated Switchgear (GIS) is crucial for power system safety, yet automatic foreign object inspection within its cavities remains challenging due to low-light conditions and strong reflections. This paper proposes a multi-modal few-shot learning framework for high-precision foreign object segmentation in GIS. To overcome imaging interference, we first establish a dual-light (visible and ultraviolet) image acquisition system and design a lightweight fusion network to adaptively integrate multi-modal features, enhancing scene representation. For the core few-shot segmentation task, we introduce a novel Multi-Similarity Guided Branch Network (MSBNet). This network employs a support-query dual-branch architecture to extract sample prototypes. It features an improved background similarity guidance mechanism to suppress base-class feature interference and a multi-similarity fusion module that synergistically integrates multi-level and multi-metric information, which significantly improves the continuity and boundary accuracy of the segmentation masks. Experiments on our GIS dataset demonstrate that, under extremely limited sample conditions, the proposed method rapidly adapts to unseen foreign object classes and substantially outperforms existing few-shot segmentation baselines.

## 1. Introduction

Gas-Insulated Switchgear (GIS) is a core component of power systems operating at high, ultra-high, and extra-high voltage levels. Its compact structure, high reliability, and strong safety performance are essential for ensuring the stable and secure operation of power grids. However, statistics indicate that nearly 40% of GIS equipment failures are attributable to internal foreign objects [1,2,3]. Under the influence of an AC electric field, these objects migrate to regions of weak electric field strength, where they can trigger partial discharge [4]. This may lead to severe systemic consequences, including busbar outages and large-scale load transfers [5,6], ultimately resulting in significant safety incidents and economic losses. Therefore, accurate detection and localization of internal foreign objects in GIS equipment and timely cleaning are key links in power grid operation and maintenance.

Traditional approaches to foreign object detection in GIS equipment often require manual entry into enclosed cavities, a process that is both time-consuming and costly. Although ultrasonic partial discharge detection and X-ray inspection have since been introduced, both methods have inherent limitations. Ultrasonic techniques suffer from poor localization accuracy due to the propagation characteristics of acoustic signals, while X-ray methods pose radiation risks and suffer from low imaging contrast. Consequently, neither approach enables reliable recognition of foreign objects.

Recently, machine vision-based detection systems integrated into intelligent inspection robots have emerged as a promising solution [7]. These systems enable non-contact, high-precision inspection, thereby improving maintenance efficiency while reducing both safety risks and resource consumption. Due to their advantages in speed, accuracy, and cost-effectiveness, machine vision methods have become an important tool for foreign object detection in GIS equipment. The integration of deep learning techniques has further enhanced their detection performance. For instance, Convolutional Neural Network (CNN)-based image classification algorithms have been applied to detect component faults [8]; improved YOLO-based object detection models have been used to recognize and roughly localize internal foreign objects [9]; and semantic segmentation algorithms have enabled pixel-level localization and segmentation of such objects [10].

**Figure 1:**
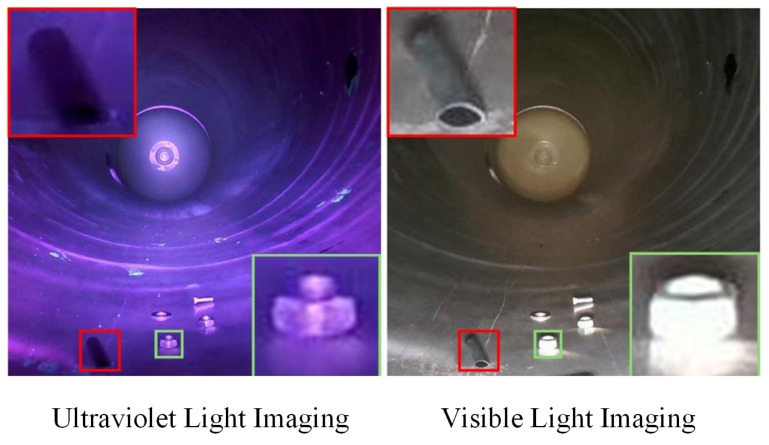
Comparison of single spectral light source imaging.

Despite the progress made by deep learning-based visual detection methods, their practical deployment still faces two key technological challenges. First, due to the enclosed nature of GIS cavities, single-spectrum illumination suffers from significant limitations, making it difficult to reliably distinguish foreign objects from the background. As illustrated in Figure 1, under visible light (400 nm–700 nm), specular reflection from metallic surfaces generates highlighted artifacts on the inner walls of the equipment, causing the edges of foreign objects to blend with the background and rendering them difficult to discern. Although ultraviolet light (<400 nm) can mitigate some reflection interference, it suffers from insufficient intensity and introduces color distortion, rendering it inadequate for reliable feature extraction. Second, samples of internal defects in GIS equipment are scarce, and acquiring large-scale annotated datasets is challenging. As a result, conventional deep learning models are often undertrained, leading to a sharp decline in segmentation performance under extremely limited sample conditions. Existing algorithms exhibit limited adaptability and generalization to unseen foreign object classes under few-shot conditions, which constrains their practicality and deployment effectiveness in real-world operation and maintenance scenarios.

Therefore, to overcome the limitations of traditional methods in multi-modal fusion and few-shot adaptation, this study proposes a few-shot learning framework that integrates dual-spectrum information. By leveraging multi-modal feature collaboration and few-shot reasoning mechanisms, the proposed framework aims to effectively address the challenges of reflection interference and data scarcity within GIS cavities. It enables robust detection and accurate segmentation of foreign objects, thereby offering a new technical pathway to enhance the practicality and reliability of intelligent GIS inspection.

The main contributions of this work are threefold:

(a) A lightweight multi-light source image fusion network is proposed. It effectively mitigates low-light and strong-reflection interference, generating high-quality, clear images of the cavity interior that serve as reliable inputs for subsequent detection tasks.
(b) A multi-similarity guided few-shot foreign object segmentation network, termed MSBNet, is designed. Through its dual-branch architecture and multi-similarity fusion mechanism, it achieves accurate and complete segmentation of unseen foreign object classes using extremely limited samples, addressing the challenge of few-shot defect detection in industrial scenarios.
(c) An end-to-end analytical framework is constructed, spanning from image enhancement to few-shot segmentation. Its effectiveness is validated on a self-built GIS dataset, demonstrating significant improvements in the robustness and practicality of internal foreign object detection under complex environmental conditions.

## 2. Related Works

Aiming at the two core challenges of complex imaging environment and scarce available defect samples in the task of internal foreign object detection in GIS equipment, this section reviews the research status of related technologies. It mainly includes two parts: image fusion technology aimed at improving image quality and few-shot semantic segmentation technology for sample-deficient scenarios.

### 2.1. Research Status of Image Fusion Technology

Image fusion aims to integrate multi-source image information to generate a single image with richer information and higher quality. Traditional methods are mostly based on mathematical tools such as multi-scale transform and sparse representation and manually designed fusion rules. Although they can retain some details, they have limitations such as complex algorithms and poor adaptive ability.

With the development of deep learning, fusion methods based on CNN have become mainstream. Early work (such as Li et al. [11]) used pre-trained networks (VGG-19) to extract features and design fusion strategies. Subsequent studies enhanced the feature extraction and reconstruction ability of networks by introducing dense connections [12], residual structures [13], two-stage training [14], and other means. Generative Adversarial Networks (GANs) have also been introduced into this field, and fusion rules are automatically learned through adversarial training (such as FusionGAN [15]), and improvements such as dual discriminator architecture [16], attention mechanism [17], and task joint learning [18] have been developed to balance information of different modalities and improve visual quality.

In recent years, Transformer has been widely used in visual tasks due to its powerful long-range dependency modeling ability and has been used for image fusion. Research work mainly focuses on how to combine the local feature extraction advantages of CNNs with the global relationship modeling ability of Transformer, such as constructing CNN–Transformer heterogeneous dual-branch networks [19] and using Swin Transformer to explore cross-modal complementarity [20]. These methods aim to more effectively fuse the salient targets of infrared images with the texture details of visible light images, improving the overall quality and task adaptability of fused images. In parallel, unsupervised learning frameworks have emerged to address the challenge of limited labeled data in industrial scenarios. VIF-Net [21] proposes an unsupervised end-to-end architecture for infrared and visible image fusion, employing a mixed loss function combining modified structural similarity (M-SSIM) and total variation (TV) to adaptively fuse thermal radiation and texture details while suppressing noise. This unsupervised paradigm is particularly relevant to GIS inspection where large-scale annotated multi-modal datasets are difficult to acquire. More recently, DSKFuse [22] introduces a passive–active distillation learning framework integrating Dynamic Sparse Transformer and Kolmogorov–Arnold Network (KAN) for lightweight multi-modal fusion. By distilling knowledge from complex teacher models while actively learning discriminative features, DSKFuse achieves state-of-the-art performance in both fusion quality and downstream tasks with substantially reduced model complexity. However, existing methods usually require a large amount of data and computing resources. How to achieve efficient, lightweight, and highly robust adaptive fusion, especially for industrial scenarios such as GIS cavities with specific interference patterns (strong reflection, low light), is still a direction to be further explored.

### 2.2. Foreign Object Segmentation in GIS Cavities

Foreign object segmentation inside GIS equipment is crucial to ensure its safe operation. Traditional methods rely on manually designed features such as edge detection and texture analysis, which have poor robustness and limited accuracy in complex and variable cavity environments.

Semantic segmentation methods based on deep learning (such as UNet and its variants) learn powerful feature representations through data-driven methods and have achieved significantly better performance than traditional methods under sufficient data conditions. For GIS scenarios, existing studies have improved segmentation accuracy by combining quaternion convolution with Gabor filters or fusing Transformer models. However, these methods heavily rely on a large amount of labeled data. In reality, the internal environment of GIS equipment is closed and detection opportunities are limited, resulting in extremely difficult collection of foreign object samples (especially newly emerging foreign object classes), and there is a serious problem of sample scarcity.

Few-shot learning (FSL) provides a solution to this problem, whose goal is to enable the model to quickly adapt to new classes with very few samples. In few-shot segmentation (FSS), as its sub-field, the core challenge is how to use a small number of support samples (with labels) to guide the segmentation of query images. Current mainstream methods are based on the ideas of metric learning and prototype learning: the pioneering work of Shaban et al. [23] proposes a dual-branch network, generating parameters for query image segmentation from the support set. SG-One [24] introduces mask average pooling to extract foreground prototypes and performs matching through spatial similarity. Subsequent works such as CANet [25] improve performance through iterative optimization, HSNet [26] uses high-dimensional convolution to handle dense feature correlations, while DENet [27] introduces an attention mechanism to estimate classifier weights to reduce computational complexity and improve efficiency. Although these methods have made progress on natural images, when directly applied to industrial scenarios such as GIS cavities with complex backgrounds, strong interference, and variable foreign object target shapes, they still face challenges such as base-class confusion, discontinuous segmentation, and sensitivity to imaging quality. Therefore, it is urgent to study specialized methods that can adapt to strong interference imaging environments and effectively use extremely few samples to achieve accurate segmentation of new class foreign objects.

In summary, existing studies have achieved fruitful results in the fields of image fusion and few-shot segmentation, but for the specific industrial application of internal detection of GIS equipment, the following key issues still need to be solved: (1) Design a lightweight and efficient network to specifically address strong reflection and low-light interference under multi-light sources, achieving reliable image enhancement. (2) Develop a robust algorithm that can overcome complex background interference and achieve accurate segmentation of new class foreign objects with extremely few samples. The work in this paper aims to address these challenges and propose a complete solution integrating image enhancement and few-shot segmentation.

## 3. Methodology

**Figure 2:**
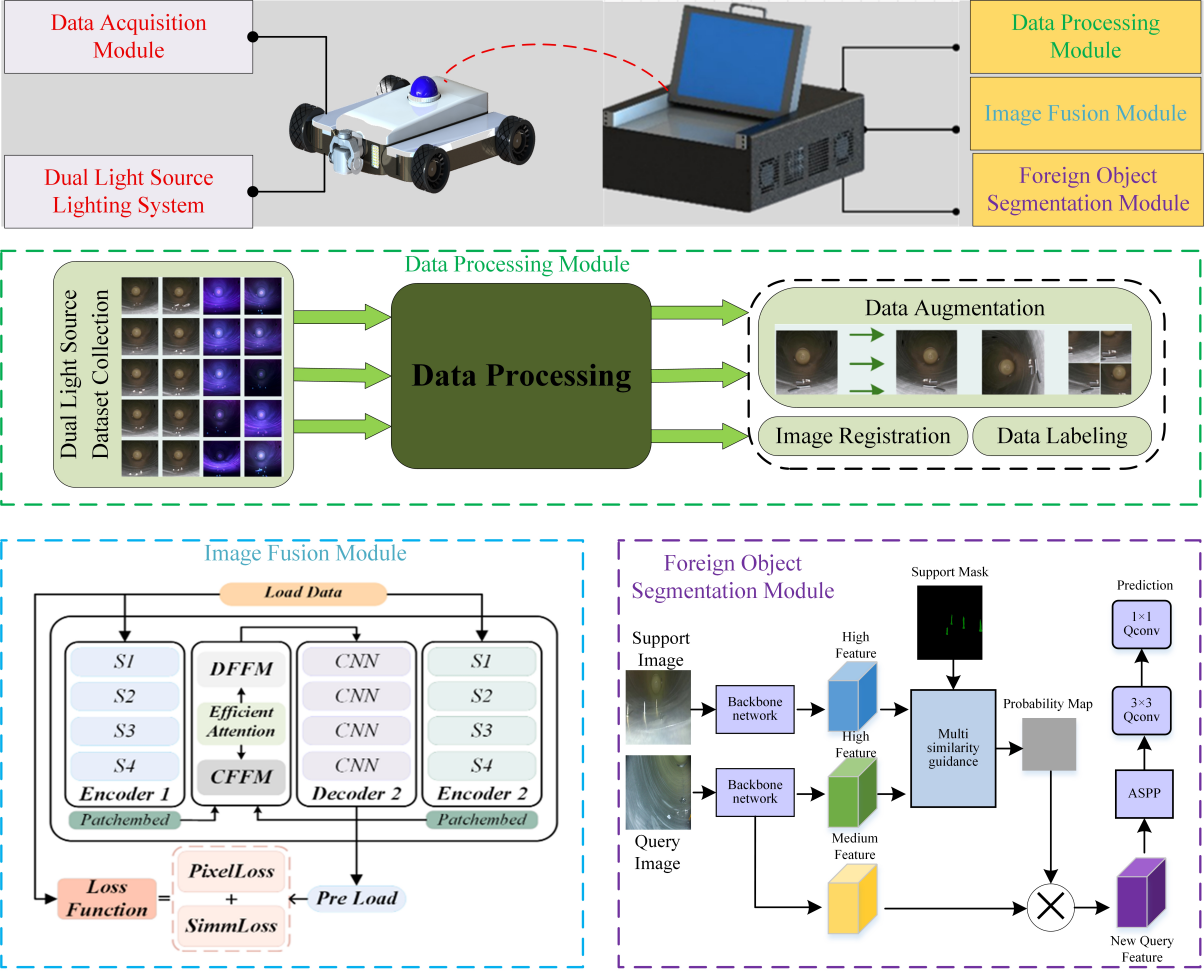
Architecture of GIS cavity internal foreign object segmentation system.

This paper constructs a complete technical system for internal foreign object detection in GIS equipment. Firstly, multi-modal cavity images are obtained through a dual-light-source data acquisition module and preprocessed. Secondly, aiming at the problem of imaging quality, a lightweight image fusion algorithm based on the attention mechanism is designed to enhance the images and generate high-quality cavity scene images. Finally, aiming at the problem of scarce samples of new class foreign objects, a multi-similarity guided few-shot segmentation network MSBNet is constructed to achieve accurate segmentation of new class foreign objects in the enhanced images. The entire system realizes a closed-loop process from data acquisition, image enhancement, to foreign object segmentation, providing a feasible technical solution for automated internal detection of GIS equipment, as shown in Figure 2.

The dual-light acquisition system comprises two LED light panels: a visible light source with a peak wavelength of 460 nm and an ultraviolet (UV) source with a peak wavelength of 365 nm. Both panels utilize OSRAM 3528 LED chips (OSRAM Opto Semiconductors GmbH, Regensburg, Germany), with illumination intensity adjustable via software-controlled voltage regulation to achieve optimal lighting conditions. Images are captured at a resolution of 1920×1080 pixels using an industrial CMOS sensor with 0.01 lux sensitivity, and visible–UV image pairs are strictly registered via hardware alignment. The 365 nm UV source effectively suppresses metallic specular reflections but exhibits lower radiant intensity than the visible panel, contributing to the insufficient illumination challenge. Furthermore, the monochromatic UV illumination combined with the sensor’s RGB color filter array introduces color distortion, motivating our dual-light fusion approach.

### 3.1. GIS Cavity Image Fusion Algorithm

#### 3.1.1. Lightweight Feature Extraction Based on Residual Feature Compensation

Feature extraction is a key step in image fusion tasks. The feature extractor of the proposed network adopts a symmetric structure, which is composed of stacked lightweight feature extraction layers to extract feature information from source images, respectively.

**Figure 3:**
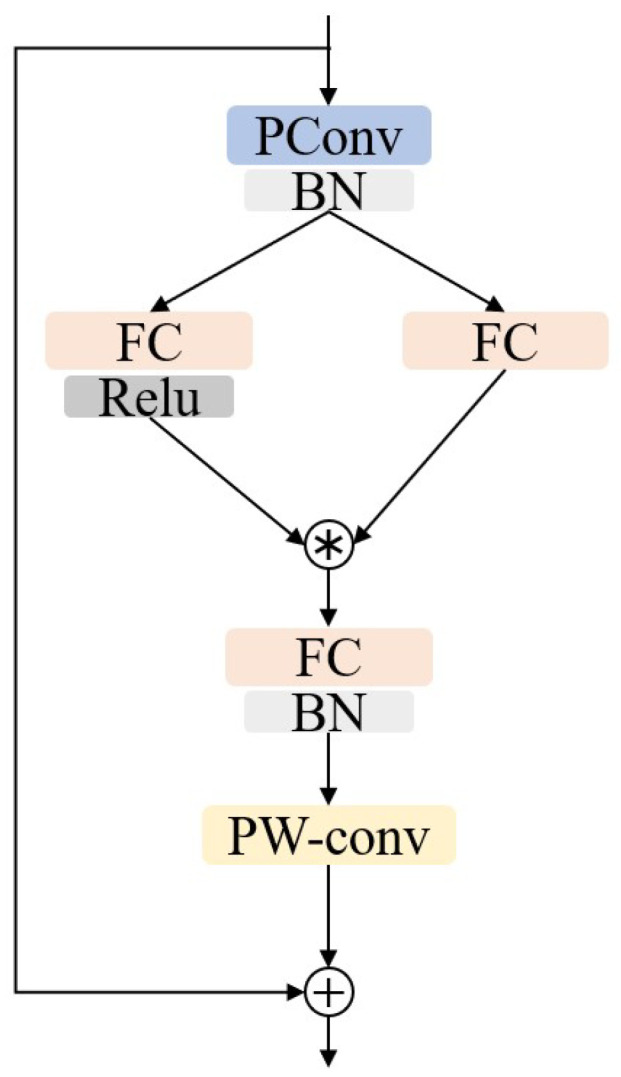
Structure diagram of lightweight feature extraction layer.

The lightweight feature extraction convolutional layer, as shown in Figure 3, is composed of a partial convolution (pconv) layer, a pointwise convolution (pwconv) layer and three Fully Connected Layers (FC).

The input image or feature is initially feature extracted through the first partial convolution layer. At the same time, to improve efficiency, batch normalization is used instead of layer normalization for normalization processing, and the calculation formula is

(1) $$F_{i}=BN\left(PConv\left(F_{input}\right)\right),i=1,2$$

where $F_{input}$ represents the input, i represents the branch number, BN represents batch normalization processing, PConv represents partial convolution operation, and the output after calculation is two branch features $F_{1}$ and $F_{2}$.

$F_{1}$ and $F_{2}$ are respectively processed by a fully connected layer and then multiplied to obtain the processed feature F, and the calculation formula is as follows:

(2) $$F=\mathrm{ReLU}\left(FC(F_{1})\right)\cdot FC\left(F_{2}\right)$$

where FC represents the fully connected layer processing, and ReLu represents activation function.

Feature F is further integrated by another fully connected layer and then input to the pointwise convolution to obtain feature F.

(3) $$F=\mathrm{PWCov}\left(\mathrm{BN}(\mathrm{FC}\left(F_{1}\right))\right)$$

where PConv represents pointwise convolution operation. Finally, the initial input is used for feature compensation of feature F after pointwise convolution through a skip connection as the final output:

(4) $$F_{\mathrm{output}}=F+F_{\mathrm{input}}$$

The features finally obtained by the feature extractor are input to the feature fusion module for further processing.

#### 3.1.2. Efficient Fusion Module Based on Cross-Attention Mechanism

**Figure 4:**
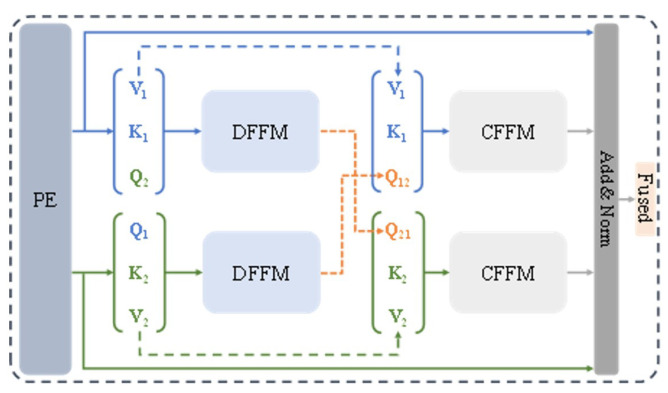
Structure diagram of efficient fusion module based on cross-attention mechanism.

The purpose of image fusion is to retain the common information in source images and supplement the mutually exclusive information, thereby outputting fused images with better visual performance. Inspired by the attention mechanism in Transformer in the field of natural language processing, this paper proposes an efficient fusion module based on a cross-attention mechanism to achieve effective fusion of dual-light-source image information, and the module structure is shown in Figure 4.

The features output by the source images after passing through the feature extractor are sent to the fusion module for feature fusion. Since the module has a symmetric structure, it will be explained from the perspective of any one modality. The input source image feature maps are respectively converted into one-dimensional feature vectors through the Patch Embedding (PE) layer and mapped into Query Matrix (Q), Key Matrix (K), and Value Matrix (V) through linear layers, and the calculation formula is as follows:

(5) $$F_{j}\left(Q_{j},K_{j},V_{j}\right)=\mathrm{Linear}\left(\mathrm{PE}(f_{j})\right)$$

where Linear represents linear mapping, PE represents patch embedding, j represents the source image category, and the outputs after the above operations are $F_{1}=\left(Q_{1},\;K_{1},\;V_{1}\right)$ and $F_{2}=\left(Q_{2},\;K_{2},\;V_{2}\right)$.

In GIS imaging, visible light provides texture but suffers from specular highlights; ultraviolet light suppresses reflections but lacks intensity. The DFFM and CFFM separate and fuse information as follows: ${\mathrm{ECFM}}_{QV}$ in Equation (6) computes the dot-product similarity between modalities to extract common structural content. Subtracting this from Vanother yields ${\mathrm{DFFM}}_{QV}$, which isolates modality-specific residuals. Adding this residual to $Q_{\mathrm{self}}$ injects complementary cues without redundancy. The symmetric CFFM (Equation (10)) then reinforces shared structural information critical for accurate localization.

**Figure 5:**
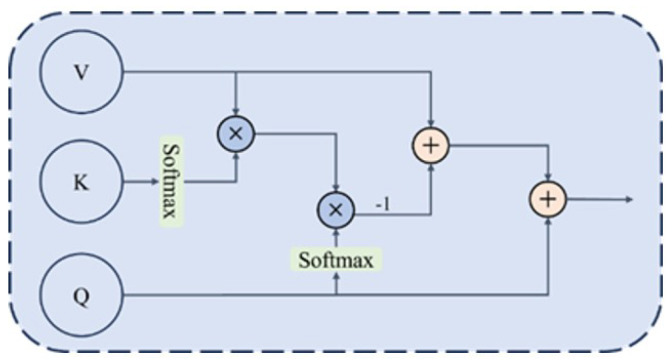
Structure diagram of the DFFM.

Then $F_{1}$ and $F_{2}$ are input to the Different Feature Fusion Module (DFFM) to calculate the mutually exclusive information between the two modalities of the source image, and the structure of the DFFM is shown in Figure 5.

To explore the common information between the two modalities of the source image, dot production is used to generate the similarity relationship between $Q_{\mathrm{self}}$ and $K_{\mathrm{another}}$ and $V_{\mathrm{another}}$ obtain the common information, then the difference information between $Q_{\mathrm{self}}$ and $V_{\mathrm{another}}$ is obtained by removing the common information, and finally, the difference information is injected into $Q_{\mathrm{self}}$ to obtain complementary features. The calculation formula is as follows:

(6) $$\begin{array}{cc} {\mathrm{ECFM}}_{QV} & =\rho_{q}\left(Q_{\mathrm{self}}\right)\left(\rho_{k}{\left(K_{\mathrm{another}}\right)}^{T}V_{\mathrm{another}}\right) \end{array}$$

(7) $$\begin{array}{cc} {\mathrm{DFFM}}_{QV} & =V_{\mathrm{another}}-{\mathrm{ECFM}}_{QV} \end{array}$$

(8) $$\begin{array}{cc} \mathrm{DFFM} & ={\mathrm{DFFM}}_{QV}+Q_{\mathrm{self}} \end{array}$$

where $\rho_{q}$ and $\rho_{k}$ respectively represent the normalized softmax operations on $Q_{\mathrm{self}}$ and $K_{\mathrm{another}}$ during efficient attention calculation. After DFFM, the two modalities of the source image can respectively obtain complementary information injection, and then they are input to the Common Feature Fusion Module (CFFM) for information integration.

**Figure 6:**
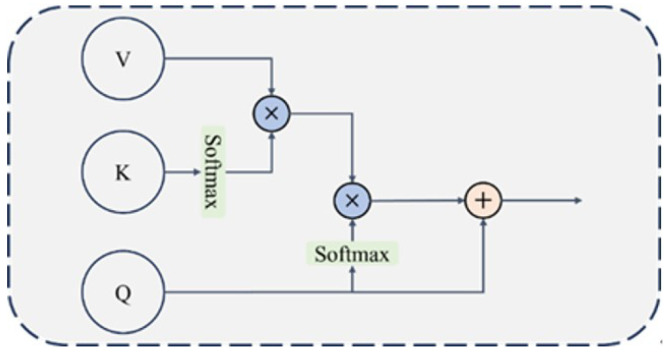
Structure diagram of CFFM.

The structure of the CFFM is shown in Figure 6. Similar to the above DFFM operation, it also uses the efficient attention mechanism to calculate common information to further supplement its own information, and the calculation formula is as follows:

(9) $$\begin{array}{cc} {\mathrm{ECFM}}_{Q_{\mathrm{add}}V} & =\rho_{q}\left(Q_{\mathrm{add}}\right)\left(\rho_{k}{\left(K_{\mathrm{self}}\right)}^{T}V_{\mathrm{self}}\right) \end{array}$$

(10) $$\begin{array}{cc} \mathrm{CFFM} & ={\mathrm{ECFM}}_{Q_{\mathrm{add}}V}+Q_{\mathrm{add}} \end{array}$$

where $Q_{\mathrm{add}}$ is the output of the DFFM, representing the feature vector of a certain modality of the source image after complementary information injection.

After the calculation of the CFFM and DFFM, the obtained feature vectors contain the information integration between the two modalities of the source image and are input to the residual connection and layer normalization (Add & Norm) layer for final information integration. At the same time, the features initially input to the fusion module are used for feature compensation and input to the Add & Norm layer through a skip connection, and finally, the feature fusion result is obtained, and the calculation formula is as follows:

(11) $$F_{\mathrm{fine}\;\mathrm{output}}=\mathrm{Add}\;\&\;\mathrm{Norm}\left(F_{1}+F_{2}+Q_{1}+Q_{2}\right)$$

$Q_{1}$ and $Q_{2}$ respectively represent the features of the two modalities after the calculation of the CFFM and DFFM.

After obtaining the fused features $F_{\mathrm{fused}}$, the image is reconstructed through four consecutive convolutional layers to output the fused image $F_{\mathrm{fuse}\;\mathrm{output}}$, and the calculation formula is as follows:

(12) $$F_{\mathrm{fused}}={\mathrm{Conv}}_{i=1}^{4}\left(F_{\mathrm{fuse}\;\mathrm{output}}\right)$$

where ${\mathrm{Conv}}_{i=1}^{4}$ represents sequential convolution operation, and i represents the i-th convolutional layer.

### 3.2. Few-Shot Foreign Object Segmentation in GIS Cavities

#### 3.2.1. Quaternion MobileNetV2 Backbone Feature Extraction Network

Quaternion MobileNetV2 is a lightweight feature extraction network specially designed for the task of foreign object segmentation in GIS cavities. This model combines the efficient inverted residual structure of MobileNetV2 with depthwise separable quaternion convolution. By replacing the depthwise separable convolution in the original model with depthwise separable quaternion convolution, it can greatly reduce the number of parameters while making full use of the advantages of quaternion algebra in multi-dimensional data representation, effectively capturing the rich structural relationships and inherent physical constraints between multiple channels of RGB images.

Traditional convolutional neural networks calculate the predicted output through forward convolution and adjust the convolution kernel through backpropagation to make the predicted output close to the real target. However, traditional real-valued convolutional neural networks use independent convolution kernels for channel-wise convolution. A good model should effectively encode the local and structural relationships of input features. For features existing in the form of quaternions, quaternion convolution maintains the independence of color space and naturally handles the coupling between channels. The multiplication of two quaternions adopts the form of a Hamilton product:

(13) $$\begin{array}{cc} Q_{1}⊗Q_{2} & =(r_{1}r_{2}-x_{1}x_{2}-y_{1}y_{2}-z_{1}z_{2})\\ \; & \;+\left(r_{1}x_{2}-x_{1}r_{2}-y_{1}z_{2}-z_{1}y_{2}\right)i\\ \; & \;+\left(r_{1}y_{2}-x_{1}z_{2}-y_{1}r_{2}-z_{1}x_{2}\right)j\\ \; & \;+\left(r_{1}z_{2}-x_{1}y_{2}-y_{1}x_{2}-z_{1}r_{2}\right)k \end{array}$$

Therefore, by embedding the Hamilton product calculation formula into the convolution calculation, the quaternion convolution process can be obtained:

(14) $$X_{ab}^{l}=\alpha \left(\sum_{c=1}^{n}\sum_{d=1}^{m}\omega_{cd}^{l}⊗X_{\left(a+c)(b+d\right)}^{l}\right)$$

where $X_{ab}^{l}$ represents the convolution output of layer l at index (a,b), w represents the convolution kernel of size n × m, and represents any activation function.

For the convenience of calculation, the convolution operation composed of equations is expressed in the form of matrix calculation, where Y, W, and X are the output matrix, weight matrix, and input matrix, respectively:

(15) $$\left[\begin{array}{c} Y_{r}\\ Y_{i}\\ Y_{j}\\ Y_{k} \end{array}\right]=\left[\begin{array}{cccc} W_{r} & W_{i} & W_{j} & W_{k}\\ -W_{i} & W_{r} & W_{k} & -W_{j}\\ -W_{j} & -W_{k} & W_{r} & W_{i}\\ -W_{k} & W_{j} & -W_{i} & W_{r} \end{array}\right]\left[\begin{array}{c} X_{r}\\ X_{i}\\ X_{j}\\ X_{k} \end{array}\right]$$

In addition, compared with real-valued convolutional neural networks, quaternion convolutional neural networks require fewer network parameters.

**Figure 7:**
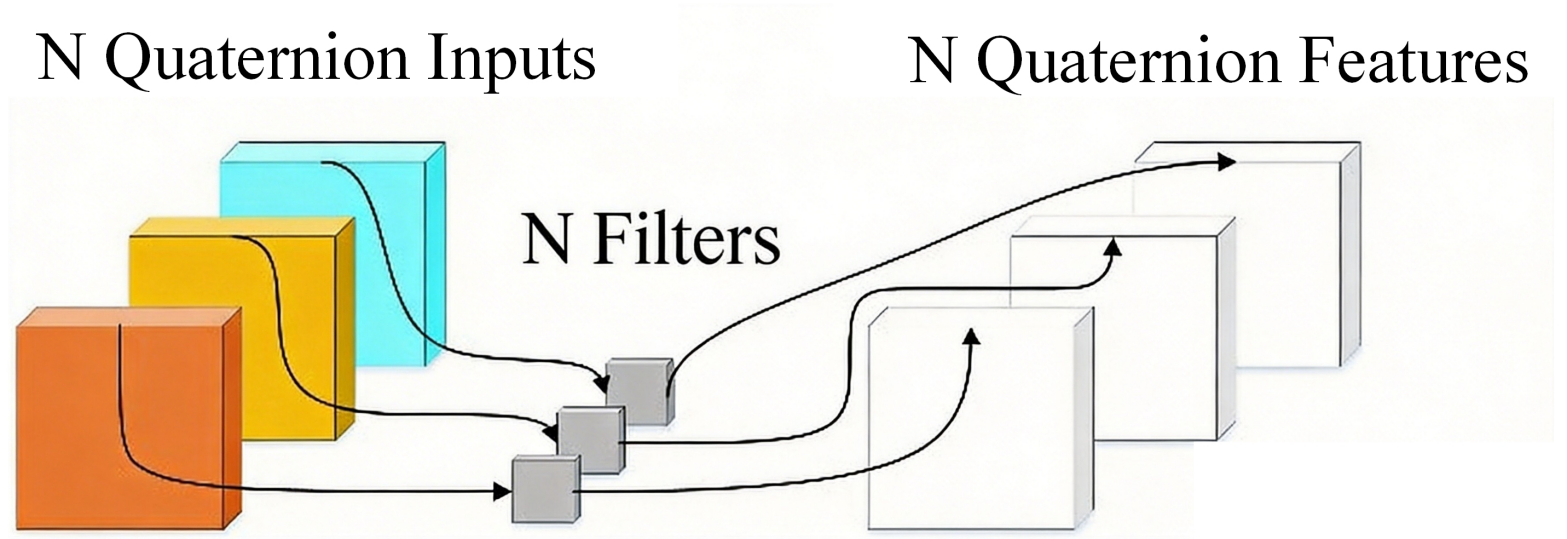
Process of depthwise separable quaternion convolution.

Furthermore, it can be extended to depthwise separable quaternion convolution. Similar to standard depthwise separable convolution, this operation also includes two parts—depthwise convolution and pointwise convolution—but its core difference lies in the different basic units of separation: the standard method takes a single channel as the unit for separation, while here, the quaternion neuron composed of four channels is taken as the basic unit. Figure 7 shows the specific calculation process of depthwise separable quaternion convolution.

In the depthwise convolution stage, each quaternion neuron composed of four-channel input is convolved with a quaternion convolution kernel respectively to generate intermediate quaternion feature maps; then, in the pointwise convolution stage, a 1 × 1 convolution with the number of convolution kernel channels equal to the number of intermediate quaternion features is used to fuse the intermediate quaternion features and map them to the final output quaternion neurons. Depthwise separable quaternion convolution performs depthwise and pointwise convolution with quaternion neurons as units, greatly reducing the number of parameters while better maintaining the internal correlation of multi-dimensional data, achieving the unity of efficiency and strong feature representation.

Standard CNNs treat RGB channels independently, ignoring inter-channel couplings caused by metallic reflections and dual-light interactions in GIS imagery. Quaternion convolution encodes four channels as a quaternion r+xi+yj+zk, with the Hamilton product imposing structured rotational relationships among channels. This yields three advantages: (i) preserved channel correlation under multi-modal fusion; (ii) 75% parameter reduction versus unconstrained 4-channel convolution (Equation (15); and (iii) natural regularization against color distortion. Depthwise separable quaternion convolution (Figure 7) extends these benefits, enabling a lightweight backbone suitable for edge deployment.

#### 3.2.2. Background Guidance Based on Cosine Similarity

In few-shot segmentation, the support image features extracted by the backbone network and the binary mask of the image are usually encoded into a representative vector to guide the query features for segmentation. Directly changing the background pixels to zero will change the statistical distribution of the support image set. Using a unified network to eliminate the background of both support images and query images at the same time will greatly increase the variance of input data. In addition, this operation will also destroy the input structure of the network, which hinders the realization of the “support-query” unified network.

Through mask average pooling operation, the features of the object content area can be extracted while ignoring the background. At the same time, mask average pooling learns better context representation without destroying the input structure of the network, enabling unified processing of the “support-query” image set.

Assuming an RGB image $I\in R^{3\times w\times h}$, its corresponding binary mask is $M\in {\left\{0,1\right\}}^{w\times h}$, where *w* and *h* are the width and height of the image, respectively. The features extracted by the network are adjusted to the same size as the binary mask through bilinear interpolation. The adjusted features are $F\in R^{c\times w\times h}$, where *c* is the number of channels of the intermediate features. By averaging the pixels on the i-th feature map, the i-th element of the representative vector can be calculated:

(16) $$v_{i}=\frac{\sum_{x=1}^{w}\sum_{y=1}^{h}M_{x,y}\cdot F_{i,x,y}}{\sum_{x=1}^{w}\sum_{y=1}^{h}M_{x,y}}$$

The purpose of few-shot segmentation is to segment the target area in the query image. Through mask average pooling operation, the representative vector of the foreground object is obtained, that is, $v=(v_{1},v_{2},\ldots ,v_{c})$, where c is the number of channels. The similarity between each pixel feature in the query feature and the foreground representative vector is calculated by similarity calculation. The larger the calculated value, the more likely the pixel belongs to the foreground category information; conversely, if the calculated value is very small, it indicates the image background. The middle-level features of the support image $I^{Q}$ extracted by the backbone network are $F^{Q}$. Then, the cosine similarity between the foreground feature vector and the query feature is calculated as follows:

(17) $$p_{x,y}=\frac{v\cdot F_{x,y}^{Q}}{{∥v∥}_{2}\cdot {∥F_{x,y}^{Q}∥}_{2}}$$

where $p_{x,y}\in \left[-1,1\right]$ is the similarity at pixel (x, y), and $F_{x,y}^{Q}$ is the feature vector of the query image at pixel (x, y). Therefore, the guidance information of the support features for the query features is integrated. The $P=\{P_{x,y}\}$ target information in the query image is guided to be segmented by multiplying the guidance map P with the query features.

Mask average pooling and cosine distance realize the object-guided segmentation of the query image by the support features. However, in GIS equipment, the few-shot segmentation network has a serious problem of false activation of base-class foreign objects. Different from other public data, the internal background of GIS equipment has high similarity and repeatability, and there is data distribution imbalance and large domain shift between foreground foreign objects, resulting in the phenomenon of false activation of base-class foreign objects, because the background similarity between images is very high. Therefore, this paper adopts the background guidance method, predicts the background by calculating the similarity between backgrounds, and then the non-background pixel part is the foreign object. Then the mask average pooling calculation formula becomes

(18) $$v_{i}=\frac{\sum_{x=1}^{w}\sum_{y=1}^{h}M_{x,y}^{\prime }\cdot F_{i,x,y}}{\sum_{x=1}^{w}\sum_{y=1}^{h}M_{x,y}^{\prime }}$$

where $M_{x,y}^{\prime }$ is the binary inverse mask of the image; that is, the background is 1 and the foreground is 0.

#### 3.2.3. Multi-Similarity Module

The feature extraction network employs multiple downsampling operations that progressively reduce spatial resolution. In standard few-shot segmentation, only the deepest feature map from the final backbone layer is used to guide query image segmentation. However, repeated downsampling causes information loss, particularly for thin or small foreign objects, resulting in discontinuous or incomplete segmentation masks.

**Figure 8:**
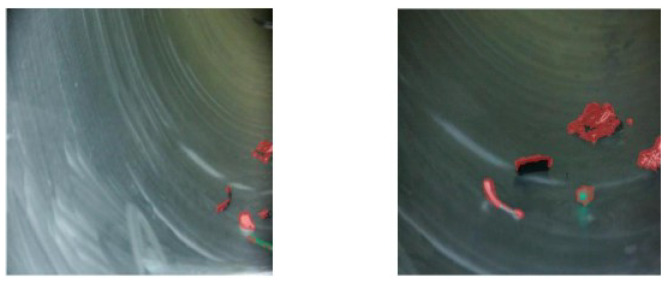
Incomplete foreign object segmentation phenomenon in few-shot segmentation network.

**Figure 9:**
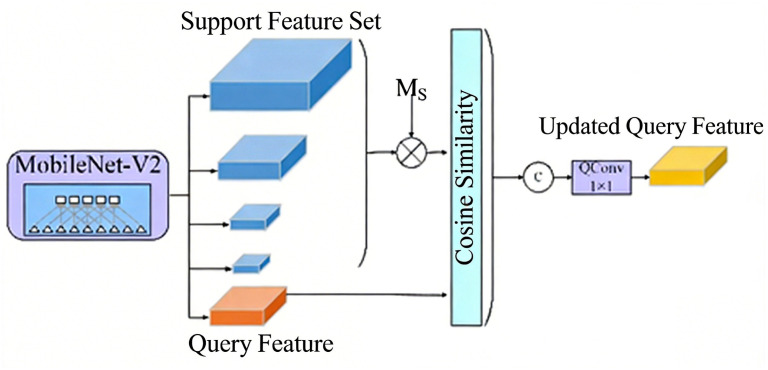
Multi-similarity guidance module.

To address this limitation, we propose a multi-similarity module that leverages intermediate features from multiple backbone stages. Specifically, we extract feature maps from the final four stages of Quaternion MobileNetV2, corresponding to downsampling factors of 4×, 8×, 16×, and 32× relative to the input resolution. These four stages are selected because they capture a comprehensive hierarchy of complementary information: the 4× downsampled features retain the richest spatial details for precise edge localization, the 8× features preserve fine-grained structural cues, the 16× features provide a balance between semantic abstraction and spatial localization, and the 32× features encode high-level semantic context essential for distinguishing foreign objects from complex GIS cavity backgrounds. For each selected stage, we compute the cosine similarity between the support prototype and the query features using Equation (17), generating a set of similarity maps $\left\{P_{4},P_{8},P_{16},P_{32}\right\}$. These maps are then fused via element-wise summation followed by a 1×1 convolution to produce the final guidance map, which is multiplied with the query features for subsequent segmentation. This multi-level fusion effectively compensates for information loss during downsampling and improves segmentation continuity, particularly for elongated or irregularly shaped foreign objects, as shown in Figure 8:

Therefore, to improve the continuity and completeness of few-shot foreign object segmentation, this paper proposes a multi-similarity module. During the process of feature resolution reduction in the feature extraction network, the cosine similarity between the feature results output by the previous convolutional layer and the query features is calculated, and the results of multiplying these similarity maps with the query features are fused to obtain new query features, as shown in Figure 9.

## 4. Experimental Section

### 4.1. Dataset and Parameter Settings

In this paper, a dual-light-source dataset is collected and constructed in real GIS equipment scenarios, which contains real-scene dual-light-source image pairs of GIS equipment with foreign objects, and has been strictly registered and aligned. The self-built dual-light-source image dataset of GIS equipment is used as the training and test data of the proposed multi-modal image fusion model to realize the training and verification of the model. The dual-light-source dataset of GIS equipment contains 752 strictly spatially registered visible–ultraviolet dual-modal image pairs. The dataset is divided into two categories: one is sufficient base-class foreign object data, and the other is new-class foreign object data. The base-class foreign object data is divided into 4 categories—screws, nuts, wire skins and background—with a total of 752 images. After being collected by the GIS robot, there are 292 new-class foreign object image targets. The total number of foreign object categories is 8. The experimental operating system in this paper is Windows 10, and the computer parameters are as follows: CPU: Intel(R) Core(TM) i7-10700k; GPU: NVIDIA GeForce RTX 3070; video memory size is 8 G. The model is built based on the Pytorch deep learning framework, and CUDA V11.1 version is installed for GPU acceleration.

To ensure unbiased evaluation across foreign object categories and prevent overfitting to specific object types, we adopt a strict leave-one-class-out cross-validation protocol for few-shot segmentation experiments. The dataset comprises 8 foreign object categories in total, with 4 base classes (screws, nuts, wire skins, background) and 4 novel classes. For each experimental run, we select one novel class as the target category for few-shot adaptation, while the remaining categories serve as training data for the base learner where applicable. Support and query images are sampled from the held-out novel class without overlap. This protocol ensures that the model is evaluated on object types completely unseen during base training, providing a rigorous assessment of few-shot generalization capability.

### 4.2. Loss Function

#### 4.2.1. Dual-Light-Source Image Fusion Loss Function

For the dual-light image fusion task, the pixel loss and structural similarity loss are set as the loss functions of the proposed network. The pixel loss directly calculates the difference in pixel values between the fused image and the target image, ensuring the intensity consistency of pixel values between them.

(19) $$L_{\mathrm{pixel}}=\omega_{\mathrm{uv}}{∥O-I_{\mathrm{uv}}∥}_{F}^{2}+\left(1-\omega_{\mathrm{uv}}\right){∥O-I_{\mathrm{rgb}}∥}_{F}^{2}$$

In the formula, $I_{\mathrm{rgb}}$, $I_{\mathrm{uv}}$, and *o*, respectively, refer to the bimodal image input pair and the fused image output; $w_{\mathrm{uv}}$ dynamically calculates the weight of the $I_{\mathrm{uv}}$ loss component.

Structural loss places greater emphasis on structural information such as edges and textures in the image, ensuring consistency in brightness, contrast, and structure between the fused image and the target image.

(20) $$L_{\mathrm{ssim}}=\omega_{\mathrm{uv}}\left[1-\mathrm{ssim}\left(O,I_{\mathrm{uv}}\right)\right]+\left(1-\omega_{\mathrm{uv}}\right)\left[1-\mathrm{ssim}\left(O,I_{\mathrm{rgb}}\right)\right]$$

Among them, ssim calculates the structural similarity between images.

The weight $\omega_{\mathrm{uv}}$ in Equations (19) and (20) is dynamically computed based on the relative illumination quality of the two source images. Specifically, we calculate the mean intensity $\mu_{\mathrm{rgb}}$ and $\mu_{\mathrm{uv}}$ for each input image pair, and define the weight as

(21) $$\omega_{\mathrm{uv}}=\frac{\mu_{\mathrm{uv}}}{\mu_{\mathrm{rgb}}+\mu_{\mathrm{uv}}}$$

This formulation adaptively assigns higher weight to the UV component when the UV image exhibits sufficient illumination and conversely relies more heavily on the visible light component when UV intensity is low. To prevent extreme weighting in dark scenes, we clip $\omega_{\mathrm{uv}}$ to the range [0.2, 0.8]. This dynamic weighting strategy ensures balanced fusion across varying lighting conditions within the GIS cavity, particularly in regions where one modality suffers from severe degradation (e.g., specular highlights in RGB or insufficient intensity in UV).

By setting two loss functions, pixel loss and structural loss, to jointly drive model training and guide the learning direction of the network, the total loss is

(22) $$L_{\mathrm{total}}=\alpha L_{\mathrm{pixel}}+L_{\mathrm{ssim}}$$

α is a balance factor.

#### 4.2.2. Foreign Object Segmentation Loss Function

In the segmentation task, the cross-entropy loss is adopted as the supervision signal to measure the pixel-level discrepancy between the predicted segmentation map and the ground truth mask. It is formulated as

(23) $$L_{\mathrm{seg}}=-\frac{1}{N}\sum_{i=1}^{N}\left[y_{i}\log\left(p_{i}\right)+\left(1-y_{i}\right)\log\left(1-p_{i}\right)\right]$$

where *N* denotes the total number of pixels, $y_{i}\in \left\{0,1\right\}$ is the ground truth label of pixel *i* (with 1 for foreground and 0 for background), and $p_{i}\in \left[0,1\right]$ is the predicted probability that pixel *i* belongs to the foreground object. This loss function effectively guides the MSBNet to learn discriminative features and achieve accurate foreign object segmentation under few-shot settings.

### 4.3. Experimental Analysis

#### 4.3.1. Qualitative and Quantitative Experimental Analyses of Image Fusion Algorithm

To fully prove the effectiveness of the proposed multi-modal image fusion model, experiments are carried out on the dual-light-source dataset of GIS equipment from two dimensions: quantitative indicators and qualitative visualization. The compared algorithms are trained in the same training environment, and their parameters are adjusted on the basis of the source code to achieve the best index results on this dataset. The quantitative and qualitative experimental results show that the proposed algorithm has better performance compared with other image fusion algorithms.

The quantitative experimental results are shown in Table 1 below. The proposed algorithm is compared with other existing advanced image fusion algorithms, where red and blue represent the optimal and sub-optimal values, respectively.

It can be seen from Table 1 that the fused images obtained by the proposed algorithm achieve the optimal values in three indicators and sub-optimal values in two indicators, which shows that the proposed algorithm has excellent performance. Specifically, the proposed algorithm achieves the optimal values in SD, EN, and SF indicators, indicating that the fused image has rich contrast, texture, and detail information. This is because the proposed fusion strategy can fully retain the texture and detail information in visible light images while obtaining the salient information in ultraviolet images; the AG and VIFF indicators achieve sub-optimal values, indicating that the fused image has good clarity and effectively retains the rich information in source images. This is because the designed feature extraction module can more effectively extract features from source images, and the proposed feature fusion strategy can also obtain and retain the rich common information in source images. Therefore, the quantitative experimental results show the excellent performance of the proposed image fusion algorithm.

**Figure 10:**
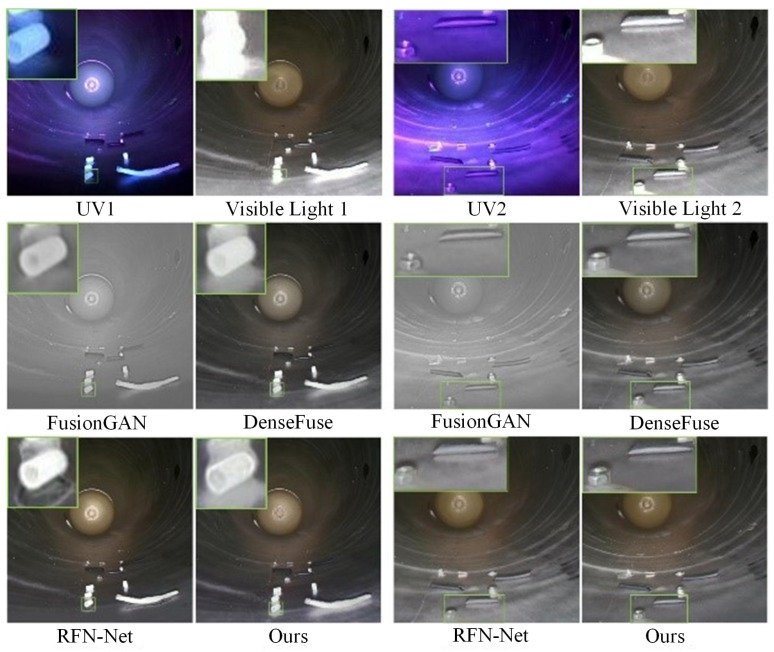
Visualization comparison results of dual-light-source dataset of GIS equipment.

To more intuitively show the quality of fused images and reflect the advancement of the proposed image fusion algorithm, two groups of images are selected from the dual-light-source dataset of GIS equipment for visualization experiments and compared with other algorithms. The visualization results are shown in Figure 10.

It can be seen from the above figure that the fused images obtained by the proposed fusion algorithm have better visual performance compared with other advanced algorithms. Specifically, the fused images of this paper have excellent performance in color and texture details and have a better weakening effect on background interference. The FusionGAN algorithm obtains the optimal AG index in quantitative experiments, but its visual performance is poor. This is because the model introduces additional noise interference during the adversarial training process, so the obtained fused images have more noise. The images of the DenseFuse algorithm have good clarity and retain texture and detail information, but their overall color is distorted and dark. This is because the fusion strategy of this algorithm is simple, resulting in insufficient color information. The overall image quality of the RFN-Nest algorithm is close to that of this paper, retaining the color, texture and detail information in source images, and can effectively weaken background interference, but it introduces new noise while weakening interference, resulting in local distortion of the image. In summary, the proposed fusion algorithm can output high-quality visualized fused images, which are more in line with the needs of visual observation.

#### 4.3.2. Ablation Experimental of Image Fusion Algorithm

Aiming at the problems of complex lighting conditions, serious background interference, and insufficient utilization of multi-modal information inside GIS equipment, this section designs a feature fusion mechanism based on an efficient attention mechanism. The above quantitative and qualitative experiments verify the superiority of the proposed feature fusion mechanism. In this section, ablation experiments are further carried out on the dual-light-source dataset of GIS equipment to prove its effectiveness. The quantitative ablation experimental results are shown in Table 2 below. It can be seen from the table that the proposed feature fusion module significantly improves the measurement of all evaluation indicators and has a positive impact on the quality of fused images.

**Figure 11:**
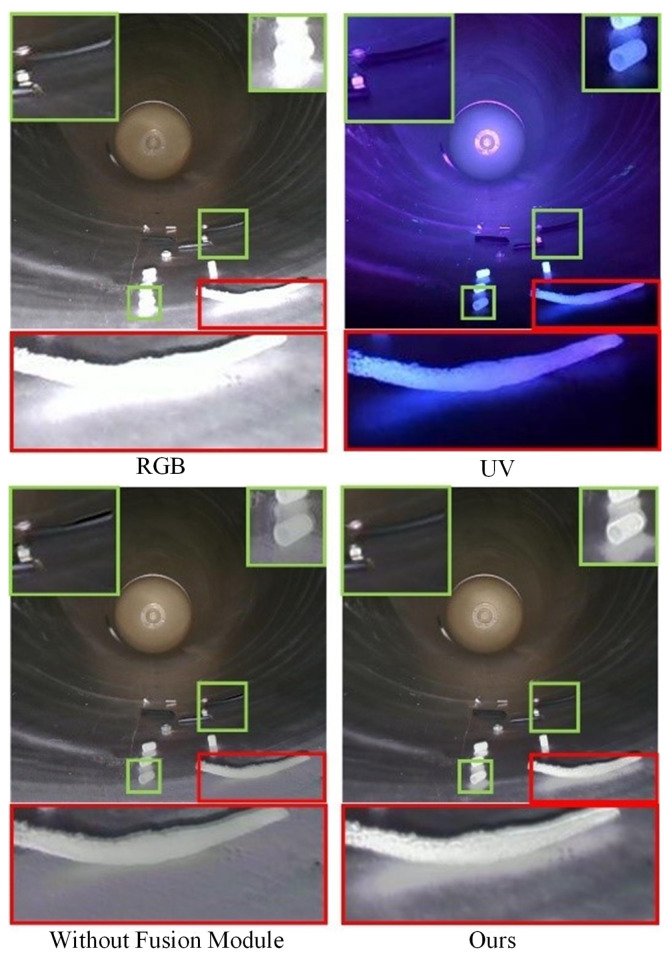
Ablation comparison results of dual-light-source dataset of GIS equipment.

In addition, to more intuitively feel the impact of the feature fusion mechanism on the quality of fused images, Figure 11 provides a set of visual ablation results. The colored areas in the figure are local magnifications to show specific detail differences.

As shown in Figure 11, when the feature fusion module is missing, the network cannot fully explore the complementary and common information in source images. From the magnified green area, it can be seen that the contrast of plastic foreign objects is insufficient, and the outline is not significant; the color information of black wires is locally distorted. From the magnified red area, it can be seen that when the fusion module is missing, the background color and foreign objects such as protective clothing have large-area color distortion and texture loss, which cannot reflect the real scene. In summary, the ablation experimental results prove the positive impact of the proposed feature fusion module on fusion performance.

To investigate whether improved fusion quality translates to better segmentation, we evaluate the proposed MSBNet using fused images generated by different fusion methods. Table 3 reports the one-shot mIoU results alongside fusion quality metrics (SD and EN). Higher SD and EN generally correlate with better segmentation accuracy, but the relationship is not strictly linear. FusionGAN yields the lowest mIoU (48.35%) despite moderate EN due to boundary-distorting adversarial artifacts. The variant without our fusion module achieves only 51.12% mIoU, confirming that the proposed cross-attention fusion contributes substantially to downstream performance. MSBNet with our full fusion method attains 59.47% mIoU, demonstrating that suppressing specular interference while preserving structural details is critical for accurate few-shot segmentation.

#### 4.3.3. Performance Comparison and Analysis of Existing Few-Shot Segmentation Algorithms

To rigorously evaluate the effectiveness of the proposed MSBNet, we conduct comprehensive comparisons with state-of-the-art few-shot segmentation methods, including SG-One [24], PFENet [29], HSNet [26], BAM [27], OCNet [30], and the recent large-scale visual prompt model VLP-SAM [31]. To ensure a fair and controlled comparison, all methods are evaluated under three distinct backbone architectures: VGG16, ResNet50, and Quaternion MobileNetV2. The comprehensive results, including parameter counts, are presented in Table 4.

As shown in the ResNet50 block of Table 4, when evaluated under the identical standard ResNet50 backbone, MSBNet achieves 60.15% mIoU in the one-shot setting and 62.34% in the five-shot setting. These results surpass the previous state-of-the-art method BAM by +1.67% and +1.31%, respectively, and also outperform the recently proposed OCNet. This consistent improvement, isolated from backbone architecture variations, confirms that the performance gains stem fundamentally from our proposed multi-similarity guidance mechanism and Background Similarity Suppression strategy rather than merely from a stronger feature extractor.

Notably, VLP-SAM, which leverages a massive vision–language backbone with over 668M parameters, achieves marginally higher accuracy (60.58% 1-shot mIoU) than our ResNet50-based MSBNet (60.15%). However, our method requires only 4.5% of VLP-SAM’s parameters while delivering comparable segmentation quality. This demonstrates that our task-specific architectural design can rival much larger general-purpose models in specialized industrial inspection scenarios.

A key contribution of this work is the integration of the lightweight Quaternion MobileNetV2 backbone, specifically designed for deployment on resource-constrained GIS inspection robots. As detailed in the third block of Table 4, MSBNet with Quaternion MobileNetV2 achieves a remarkable 59.47% one-shot mIoU and 61.56% five-shot mIoU. This performance is competitive with ResNet50-based MSBNet (retaining approximately 98.8% of the accuracy) while utilizing only 6.61M parameters. Compared to VGG16-based counterparts with over 140M parameters, our lightweight solution achieves substantially higher accuracy with 95% fewer parameters.

**Figure 12:**
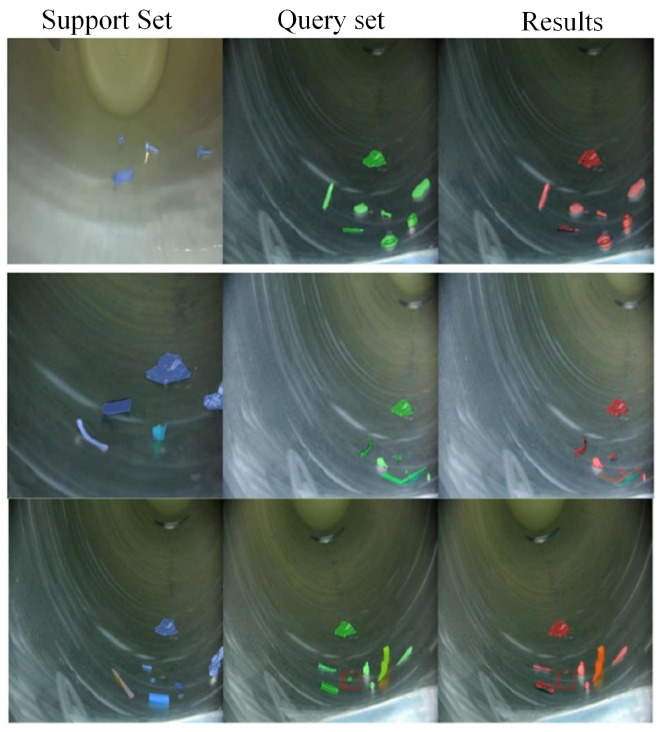
Experimental results of few-shot foreign object segmentation for GIS under 1-shot.

Figure 12 presents the visualization results of MSBNet under the one-shot setting. The predicted segmentation masks closely align with the ground truth annotations, demonstrating precise boundary delineation and complete object coverage. While occasional failures occur when foreign objects exhibit extreme visual similarity to GIS cavity structures, the multi-similarity module significantly mitigates the discontinuity and incompleteness issues commonly observed in few-shot segmentation networks.

In summary, the comprehensive experimental results validate that MSBNet not only achieves state-of-the-art accuracy across diverse backbone configurations but also offers an exceptional accuracy–efficiency trade-off when paired with the Quaternion MobileNetV2 backbone. This dual advantage renders the proposed framework highly practical for real-world deployment in automated GIS inspection systems.

To quantitatively validate the lightweight nature of the proposed framework, we conduct a detailed comparison of model complexity between MSBNet and the state-of-the-art baseline BAM [27]. Since the lightweight characteristic of MSBNet primarily stems from its feature extraction backbone—Quaternion MobileNetV2—we compare both methods under two backbone configurations: the standard ResNet50 and our proposed Quaternion MobileNetV2.

As shown in Table 5, under the ResNet50 backbone, MSBNet and BAM exhibit comparable complexity. However, with Quaternion MobileNetV2, MSBNet requires only 6.61M parameters and 15.8G FLOPs—reductions of 78.0% and 76.4%, respectively, compared to its ResNet50 counterpart—while achieving 75.4 FPS. Notably, this lightweight variant retains 98.9% of the ResNet50 version’s accuracy (59.47% vs. 60.15% 1-shot mIoU) and outperforms BAM with the same backbone by +5.45%. These results demonstrate that MSBNet achieves an optimal accuracy–efficiency trade-off suitable for real-time deployment on resource-constrained GIS inspection robots.

#### 4.3.4. Ablation Experimental of Few-Shot Foreign Object Segmentation Algorithm

The experiments in Table 6 are the results obtained from one-shot experiments. Among them, BS is background similarity, and MS is the multi-similarity guidance module. The experimental data in the first row is obtained by extracting deep support features from Quaternion MobileNet-V2 and guiding the similarity of its middle query features. It can be seen that after adding background similarity, the mIoU and FB-IoU of the algorithm have been greatly improved, alleviating the false activation phenomenon of base-class foreign objects in GIS equipment. The multi-similarity module supplements the information loss caused by multiple downsampling operations that reduce resolution in the backbone network Quaternion MobileNet-V2, further improving mIoU and FB-IoU. However, the multi-similarity module only supplements the lost information and does not solve the problem of false activation of base-class foreign objects. Therefore, adding only the multi-similarity module does not significantly improve the algorithm.

To quantitatively validate the contribution of multi-modal fusion to downstream few-shot segmentation, we compare MSBNet (Quaternion MobileV2 backbone) trained on three different input configurations: (i) visible light only (RGB), (ii) UV only, and (iii) our fused dual-light images. All experiments follow the identical one-shot protocol. The results are summarized in Table 7.

The visible-only configuration suffers from specular highlights on metallic surfaces, causing false negatives where foreign objects blend into reflections. The UV-only configuration mitigates highlights but exhibits poor intensity and color distortion, resulting in the lowest overall accuracy. Our fused images effectively combine the texture detail of visible light with the reflection suppression of UV, yielding a substantial +8.24% absolute improvement over the visible-only baseline and +12.62% over UV-only. This ablation quantitatively demonstrates that multi-modal fusion is a critical component of the overall framework, directly addressing the imaging challenges inherent to GIS cavity inspection.

#### 4.3.5. Failure Case Analysis

**Figure 13:**
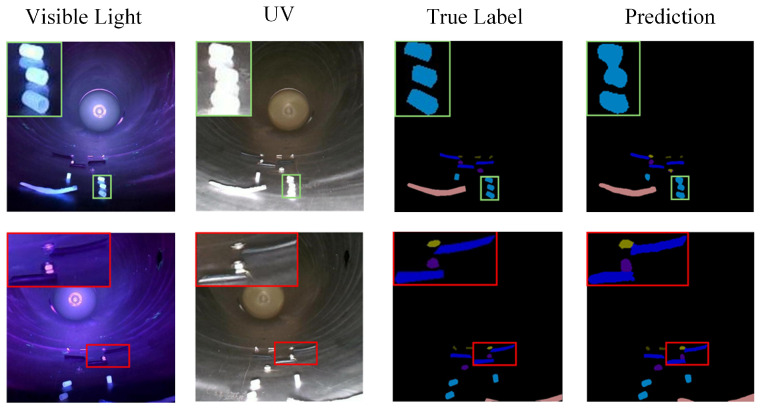
Representative failure cases of MSBNet under 1-shot setting.

We identify two representative failure modes of MSBNet, as illustrated in Figure 13. First, when directional illumination casts shadows adjacent to foreign objects, MSBNet may merge the shadow with the target, as low-intensity shadow regions resemble dark foreign materials. Second, when foreign objects share high visual similarity with metallic cavity walls, background regions may be falsely activated as foreground, particularly under illumination or viewpoint shifts between support and query images. These failures suggest that purely appearance-based similarity may be insufficient under extreme conditions. Future work could incorporate geometric priors or illumination-invariant features to address these limitations.

## 5. Conclusions

This paper presents a multi-modal few-shot segmentation framework to tackle the challenges of low-light interference and scarce samples in GIS foreign object segmentation. The main contributions are threefold. First, a complete inspection pipeline is established, integrating dual-light-source acquisition, image fusion, and segmentation, along with a registered visible–ultraviolet dataset. Second, a lightweight attention-based fusion network is proposed to effectively mitigate complex lighting interference, generating high-quality cavity images. Third, a Multi-Similarity Guided Branch Network (MSBNet) is designed, which leverages a quaternion MobileNetV2 backbone, a background similarity guidance mechanism, and a multi-similarity fusion module to achieve accurate and continuous segmentation of novel foreign objects with extremely limited samples. Extensive experiments demonstrate that our method outperforms mainstream algorithms in both image fusion quality and few-shot segmentation accuracy. Future work will explore unsupervised multi-modal GIS foreign object segmentation techniques to enhance practical deployability. We also plan to extend the framework to other industrial inspection tasks, such as surface defect detection on insulators or transformers, to validate its generalizability.

## Figures and Tables

**Table 1:** Comparison of quantitative indicators for GIS dual-light-source dataset. (AG: Average Gradient, EN: Entropy, SD: Standard Deviation, SF: Spatial Frequency, VIFF: Visual Information Fidelity for Fusion, and Qabf: Quality Assessment based on Fusion). Bold values indicate the best performance.

Method	AG	EN	SD	SF	VIFF	Qabf
DenseFuse [11]	1.179	6.420	8.666	0.009	0.607	**0.344**
FusionGAN [28]	**2.072**	5.933	5.736	0.001	**0.652**	0.302
RFN-Nest [24]	1.146	6.237	8.254	0.009	0.613	0.279
**Ours**	1.34	**6.622**	**10.377**	**0.0096**	0.641	0.285

**Table 2:** Detailed ablation study metrics for the dual-light-source GIS equipment dataset. Bold values indicate the best performance.

Method	Evaluation Metric					
	AG	EN	SD	SF	VIFF	Qabf
Missing feature fusion module	1.203	6.239	8.284	0.007	0.577	0.254
**Ours**	**1.383**	**6.622**	**10.377**	**0.009**	**0.641**	**0.284**

**Table 3:** Impact of fusion quality on MSBNet segmentation performance. Bold values indicate the best performance.

Fusion Method	SD	EN	1-Shot mIoU (%)
FusionGAN	5.736	5.933	48.35
RFN-Nest	8.254	6.237	54.68
DenseFuse	8.666	6.420	55.21
w/o Fusion Module	8.284	6.239	51.12
Ours	**10.377**	**6.622**	**59.47**

**Table 4:** Comparison with existing advanced algorithms (metrics: mIoU %). Bold values indicate the best performance.

Backbone	Method	Params (M)	1-Shot	5-Shot	Average
VGG16	SG-One [24]	138.1	39.56	43.66	41.61
	PFENet [29]	140.5	48.85	56.03	52.44
	HSNet [26]	141.8	51.54	57.00	54.27
	BAM [27]	143.2	52.45	57.57	55.01
	OCNet [30]	143.1	54.60	59.32	56.96
	MSBNet (Ours)	142.5	55.28	60.76	57.72
ResNet50	SG-One [24]	25.6	44.62	49.31	46.97
	PFENet [29]	28.0	53.15	54.39	53.77
	HSNet [26]	29.3	56.89	58.60	57.75
	BAM [27]	30.7	58.48	61.03	59.76
	OCNet [30]	30.1	59.52	61.93	60.92
	VLP-SAM [31]	668.7	60.58	62.30	61.44
	MSBNet (Ours)	30.0	60.15	62.34	61.25
Quaternion MobileV2	SG-One [24]	2.2	37.85	42.10	39.98
	PFENet [29]	4.6	49.23	52.45	50.84
	HSNet [26]	5.9	52.78	56.12	54.45
	BAM [27]	7.3	54.02	57.89	55.96
	OCNet [30]	6.3	57.32	60.62	58.97
	**MSBNet (Ours)**	6.6	**59.47**	**61.56**	**60.52**

**Table 5:** Computational complexity comparison between MSBNet and BAM under different backbones.

Method	Backbone	Params (M)	FLOPs (G)	FPS
BAM [27]	ResNet50	30.7	68.4	31.2
MSBNet (Ours)	ResNet50	30.0	67.1	32.5
BAM [27]	Quaternion MobileV2	7.31	18.2	68.7
MSBNet (Ours)	Quaternion MobileV2	6.61	15.8	75.4

**Table 6:** Results of ablation experiment. (× indicates the module is not used; ✓ indicates the module is included. Bold values denote the best performance).

+BS	+MS	mIoU	FB-IoU
×	×	51.12	74.62
✓	×	56.39	76.15
×	✓	51.61	74.67
✓	✓	**59.47**	**79.40**

**Table 7:** Impact of input modality on 1-shot segmentation performance. (Bold values indicate the best performance).

Input Modality	1-Shot mIoU (%)	Relative Gain
Visible light only	51.23	—
UV only	46.85	−4.38
**Fused (Ours)**	**59.47**	**+8.24**

## Data Availability

The original contributions of this study are included in the article; further inquiries can be directed to the corresponding author.
